# A germline variant N375S in *MET* and gastric cancer susceptibility in a Chinese population

**DOI:** 10.7555/JBR.26.20110087

**Published:** 2012-03-29

**Authors:** Yao Liu, Qin Zhang, Chuanli Ren, Yanbing Ding, Guangfu Jin, Zhibin Hu, Yaochu Xu, Hongbing Shen

**Affiliations:** a Department of Epidemiology and Biostatistics, MOE Key Laboratory of Modern Toxicology, School of Public Health, Nanjing Medical University, Nanjing, Jiangsu 210029, China;; b Medical Laboratory, Northern Jiangsu People's Hospital, Yangzhou, Jiangsu 225001, China;; c Department of Gastroenterology, Yangzhou First People's Hospital, Yangzhou, Jiangsu 225009, China;; d Section of Clinical Epidemiology, Jiangsu Key Laboratory of Cancer Biomarkers, Prevention and Treatment, Cancer Center, Nanjing Medical University, Nanjing, Jiangsu 210029, China.

**Keywords:** MET, germline variation, gastric cancer, susceptibility

## Abstract

MET tyrosine kinase and its ligand, hepatocyte growth factor (HGF), play a pivotal role in the activties of tumor cells. A germline missense variant in exon 2 of the *MET* gene, N375S (rs33917957 A>G), may alter the binding affinity of MET for HGF and thus modify the risk of tumorigenesis. In this study, we performed a case-control study to assess the association between N375S and gastric cancer risk in 1,681 gastric cancer cases and 1,858 cancer-free controls. Logistic regression analysis was applied to estimate crude and adjusted odds ratios (ORs) and 95% confidence intervals (CIs) for the associations between genotypes and gastric cancer risk. We found that *MET* N375S variant genotypes (NS/SS) were associated with a significantly decreased risk of gastric cancer (OR = 0.78, 95% CI = 0.63-0.96, *P* = 0.021) compared with the wildtype homozygote (NN). The finding indicates that this germline variant in *MET* may decrease gastric cancer susceptibility in Han Chinese.

## INTRODUCTION

Although the incidence of gastric cancer decreased in the past decades, gastric cancer remains the fourth most common cancer and the second leading cause of cancer related death worldwide. About half of global gastric cancer cases occur in China, and the incidence and mortality rates (per 100,000 persons) were 42.4 and 28.1 for men and 18.3 and 13.0 for women, respectively.[1] *Helicobacter pylori* (*H. pylori*) infection has been established as a risk factor in stomach carcinogenesis[2]. Genetic factors may also play a critical role in the development of gastric cancer[3].

MET, a tyrosine kinase, and its ligand, hepatocyte growth factor (HGF), play a pivotal role in tumor cell proliferation, survival, and metastasis[4],[5]. Kunlyasu *et al*.[6] reported that MET was overexpressed in about half of gastric cancer tissues. Further investigations revealed that MET was more frequently overexpressed in diffuse gastric cancer[7]. *MET* overexpression was correlated with gastric tumor invasion and lymph node and liver metastasis[8],[9]. In addition to aberrant upregulation, mutations of *MET* were also detected in gastric cancer and they may result in the activation of cell signaling transduction pathways. Lee *et al*.[10] reported that a germline juxtamembrane mutation, P1009S, showed an increased and sustained tyrosine phosphorylation of the protein compared with the wildtype. There has not yet been any study on the relationship between germline variations of *MET* and gastric cancer risk.

We surveyed nonsynonymous variations in *MET* exons with the criteria of minor allele frequency (MAF) > 0.05 in Han Chinese and found only one variation in codon 375, N375S (rs33917957). Thus, we performed a case-control study including 1,681 gastric cancer cases and 1,858 controls to test the association between N375S and risk of gastric cancer.

## MATERIALS AND METHODS

### Study subjects

This case-control study was approved by the Insti-tutional Review Board of Nanjing Medical University. As described previously[11], newly-diagnosed gastric cancer patients were recruited from the cities of Yang-zhong and Yixing between January 2004 and July 2005 and the cities of Yangzhou and Nanjing between October 2006 and June 2010 in Jiangsu province, China. The criteria for recruitment of gastric cancer patients were as follows: 1) if these subjects were self-reported Han Chinese; 2) if they had resided in these above mentioned cities for at least 5 years; 3) if they were newly histopathologically diagnosed as primary gastric cancer; 4) if they had no previous malignancy in any other organs; 5) if they received no previous anti-tumor therapy before recruitment, including chemotherapy, and radiotherapy. As a result, a total of 1,681 gastric cancer patients were included in this study with a response rate of 87.3%. Based on the Lauren classification[12], most gastric cancer cases were divided into two subgroups, the intestinal type and diffuse type, and those with the mixed type or whose cancer type was difficult to be determined were denoted as the unclassified type.

Additionally, 1,858 controls were randomly selected from a pool of more than 40,000 cancer-free individuals who participated in the community-based screening program for non-infectious diseases conducted in Jiangsu province, with an overall response rate of 83.8%. All controls were frequency-matched to gastric cancer cases by age (5-year interval), gender and residential areas (city).

After written informed consent was obtained, each participant was surveyed by a structured questionnaire to collect information on demographic data and envir-onmental exposure history. Individuals who smoked one cigarette per day for over one year were defined as smokers, and those who consumed three or more alco-hol drinks a week for over six months were categorized as alcohol drinkers[12]–[14]. After the interview, about 5-mL venous blood sample was collected from each participant.

### Genotyping

Genomic DNA was extracted from a leukocyte pel-let by the traditional proteinase K digestion method and followed by phenol-chloroform extraction and ethanol precipitation. The germline variation, N375S (rs33917957 A>G), was genotyped using the *Taq*Man allelic discrimination assay on an ABI 7900 system (Applied Biosystems, CA, USA). The primers and probes were as follows. Primers: sense, 5′-TGCATTCCCTATCAAATATGTCAAC-3′, antisense, 5′-GCTGGAGACATCTCACATTGTTTT-3′; Probes: allele A, FAM-CTTCTTCAACAAGATC-MGB, allele G, HEX-TTCTTCAGCAAGATC-MGB. Genotyping was performed blind without knowing the subjects' case or control status. Two negative controls in each 384-well plate were used for quality control and 5% samples were randomly selected to repeat the procedure, yielding a 100% concordance rate.

### Statistical analysis

We used the Student's *t*-test (for continuous vari-ables) and χ^2^ test (for categorical variables) to detect the differences in demographic characteristics and genotype frequencies of N375S between the cases and controls. The goodness-of-fit χ^2^ test was used to evaluate Hardy-Weinberg equilibrium (HWE) in the control subjects. Logistic regression analysis was applied to estimate crude and adjusted odds ratios (ORs) and 95% confidence intervals (CIs) for the associations between genotypes and gastric cancer risk. Heterogeneity of associations between subgroups was analyzed by the Chi-square-based Q test. All of the statistical analyses were performed with Stat-istical Analysis System software (Version 9.1.3; SAS Institute, Cary, NC, USA). All tests were two-sided and the α was set at 0.05.

**Table 1:** Distribution of alleles and genotypes of N375S and their association with gastric cancer risk

Genotype	Case (*n*=1,633)		Control (*n*=1,830)		Crude OR (95%CI)	Adjusted OR (95%CI)^a^	*P*^a^
	N	%	N	%			
NN	1,463	89.6	1,593	87.0	1.00	1.00	
NS	166	10.2	230	12.6	0.79(0.64-0.97)	0.79(0.64-0.97)	
SS	4	0.2	7	0.4	0.62(0.18-2.13)	0.63(0.18-2.15)	
NS/SS	170	10.4	215	13.0	0.78(0.63-0.96)	0.78(0.63-0.96)	0.021
Per allele					0.79(0.64-0.96)	0.79(0.64-0.96)	0.020

^a^Adjusted for age, gender, smoking and drinking status. CI: confidence interval; OR: odds ratio.

## RESULTS

The demographic characteristics of 1,681 gastric cancer cases and 1,858 controls were summarized in our previous study[13], The genotyping call rate for N375S was 97.85% and the successfully genotyped cases and controls were 1,633 and 1,830, respectively. The genotype distributions in gastric cancer cases and cancer-free controls are shown in ***Table 1***. Briefly, among the 1,681 gastric cancer cases, 1,070 (63.6%) were classified as the intestinal type, 312 (18.6%) as the diffuse type, and 299 (17.8%) as unclassified. Among them, 822 (48.9%) had cardiac gastric cancer, and 725 (43.1%) had non-cardiac gastric cancer and gastric cancer in 134 (8.0%) patients was unclassified. There were no significant differences in the distribution of age, gender, smoking and drinking status between cases and controls (*P* = 0.884, 0.245, 0.198 and 0.072, respectively).

The observed genotype frequencies among the controls were in HWE (*P* = 0.851). In the logistic regression analyses, the N375S variant was associated with significantly decreased gastric cancer risk. Com-pared with individuals carrying the one word NN gen-otype in codon 375, those with NS and SS genotypes had decreased gastric cancer risk with adjusted ORs of 0.79 (95% CI = 0.64-0.97) and 0.63 (95% CI = 0.18-2.15). The variant genotypes NS/SS showed significantly decreased gastric cancer risk by 22% (Adjusted OR = 0.78, 95% CI = 0.63-0.96, *P* = 0.021) and 21% (Adjusted OR = 0.79, 95% CI = 0.64-0.96, *P* = 0.020) in the dominant and additive model, respectively.

In the stratified analysis, the variant genotypes NS/SS were associated with significantly decreased risk in non-cardiac or diffuse gastric cancer (***Table 2***). However, no significant heterogeneity was detected for the associations among the subgroups, implying independent genetic effect.

**Table 2:** Stratified analysis of N375S and gastric cancer risk

	Case (*n*=1,633)		Control (*n*=1,830)		Adjusted OR (95%CI)^a^	*P*^b^
	NN	NS/SS	NN	NS/SS		
Age (y)						
≤60	1,625(89.7)	72(10.3)	675(87.1)	100(12.9)	0.77(0.56-1.06)	0.972
> 60	1,838(89.5)	98(10.5)	918(87.0)	137(13.0)	0.79(0.60-1.04)	
Gender						
Male	1,082(89.8)	123(10.2)	1,156(87.8)	160(12.2)	0.82(0.64-1.05)	0.497
Female	1,381(89.0)	47(11.0)	437(85.0)	77(15.0)	0.71(0.48-1.05)	
Smoking status						
No	1,712(88.6)	92((11.4)	878(87.0)	131(13.0)	0.87(0.66-1.16)	0.571
Yes	1,643(89.8)	73(10.2)	715(87.1)	106(12.9)	0.77(0.56-1.06)	
Drinking status						
No	1,976(88.7)	124(11.3)	1,099(86.3)	175(13.7)	0.80(0.63-1.02)	0.738
Yes	1,377(90.2)	41(9.8)	494(88.8)	62(11.2)	0.86(0.57-1.31)	
Histological type						
Intestinal	1,924(89.0)	114(11.0)	1,593(87.0)	237(13.0)	0.83(0.65-1.05)	0.290
Diffuse	1,274(91.3)	26(8.7)	1,593(87.0)	237(13.0)	0.64(0.42-0.98)	
Tumor site						
Cardia	1,709(89.2)	86(10.8)	1,593(87.0)	237(13.0)	0.81(0.62-1.05)	0.621
Non-cardia	1,636(90.1)	70(9.9)	1,593(87.0)	237(13.0)	0.75(0.57-0.99)	

Individuals smoking one cigaretts per day for over one year were defined as smokers. Individuals consumed three or more alcohol drinks per week for over six months were defined as drinkers.

^a^Adjusted for age, sex, smoking status and drinking status (excluded the stratified factor in the first four strata).

^b^*P* for heterogeneity. CI: confidence interval; OR: odds ratio.

[*n*(%)]

## DISCUSSION

Genetic variations may modify individuals' susce-ptibility to gastric cancer. It is of great importance to identify genetic variations that are associated with gastric cancer risk and potentially useful in gastric cancer risk prediction. The MET signaling pathway plays a critical role in various cancers, and the disruption of the balance of MET and HGF has been implicated in the etiology and progression of multiple cancers, including gastric cancer[6],[7]. In this study, we found that a germline variation in *MET*, N375S, was associated with a significantly decreased gastric cancer risk.

N375S is located at exon 2 of *MET*, which corres-ponds to the semaphorin domain of the MET protein, which is the ligand binding region, and is highly con-served in mammalians based on the UCSC data (http://genome.ucsc.edu/). Structure analysis indicated that N375 forms two potential hydrogen bonds, whereas S375 modeling structure retains only one hydrogen bond, which therefore weakens ligand binding affi-nity[15]. Given that the HGF/MET pathway is highly activated in gastric cancer[6]–[9], it is biologically plau-sible that the MET S375 variant with a low affinity for HGF may reduce gastric cancer risk, which is con-sistent with our findings. Wu *et al*.[7] reported that MET was more frequently overexpressed in diffuse gastric cancer, indicating that the HGF/MET pathway was hyper-activated in diffuse gastric cancer. Our study showed that the protective effect of the variant genotype of N375S was more evident in diffuse gas-tric cancer.

Although we have a large sample size to detect the effect of MET N375S on gastric cancer susceptibility, limitation does exist in this study. The data of *H. pylori* infection was not available in this study; thus, it is difficult for us to adjust the potential confounding bias from *H. pylori* infection or to evaluate the potential gene-environment interaction.

In summary, this study, with a relatively large po-pulation, provides the first evidence that a germline missense variation in *MET*, N375S, may decrease susceptibility to gastric cancer in Han Chinese. Fur-ther studies incorporating diverse populations and fun-ctional assays are warranted to validate and expand our findings.
